# Weight stigma in the COVID-19 pandemic: a scoping review

**DOI:** 10.1186/s40337-022-00563-4

**Published:** 2022-03-26

**Authors:** Patricia Fortes Cavalcanti de Macêdo, Carina Marcia Magalhães Nepomuceno, Nedja Silva dos Santos, Valterlinda Alves de Oliveira Queiroz, Emile Miranda Pereira, Lucineide da Conceição Leal, Lígia Amparo da Silva Santos, Leonardo Fernandes Nascimento, Poliana Cardoso Martins, Mônica Leila Portela de Santana

**Affiliations:** 1 School of Nutrition, Federal University of Bahia, Salvador, Brazil; 2 Metropolitan Union for the Development of Education and Culture (UNIME), Psychology Course, Salvador, Bahia Brazil; 3 Faculty of Social Sciences, Federal University of Bahia, Salvador, Brazil

**Keywords:** Weight prejudice, Feeding and eating disorders, Quarantine

## Abstract

**Background:**

Weight stigma is a phenomenon associated with adverse behavioural and psychological consequences. Although experts suggest that its increase during the COVID-19 pandemic may be associated with worse health outcomes for people with obesity, a thorough analysis of the main findings and gaps is still needed when relating to this subject.

**Objective:**

We aim to answer three questions: (1) How does weight stigma manifest in the COVID-19 pandemic? (2) How can weight stigma affect people with overweight or obesity in times of COVID-19? (3) What are the perceptions and experiences of weight stigma during the pandemic in individuals who experience overweight or obesity?

**Methods:**

We conducted a scoping review of studies addressing weight stigma and the COVID-19 pandemic in electronic databases (Medline/PubMed, CINAHL, Embase, PsycInfo, BVS/Lilacs, Scopus, Web of Science, Google Scholar, and OpenGrey) published until 10th August 2021. All relevant studies were reviewed in full by two researchers. In addition, a narrative synthesis of the data was performed.

**Results:**

The results included 35 studies out of 8,090 records and identified 14 original research publications, 15 text and opinion papers, and 6 narrative reviews. The results revealed the presence of weight stigma in the media, healthcare settings, interpersonal relationships, and public campaigns during the COVID-19 pandemic. The evidence of increasing weight stigma in the COVID-19 outbreak is limited, though. Many weight discrimination consequences were described during this time, such as impairment in accessing healthcare, worst COVID-19 outcomes, and maladaptive eating. However, only maladaptive behaviours and decline in mental health outcomes were demonstrated empirically in all age groups. This effect occurred regardless of body mass index, but people with high body weight were more likely to experience weight stigma. For some people with obesity, weight stigma in the pandemic has made activities of daily routine difficult.

**Conclusions:**

The results suggest that weight stigma in the COVID-19 pandemic occurs in several settings; moreover, although weight discrimination impacts mental health, whether before or during the pandemic, this influence between the pandemic and pre-pandemic scenario is still unclear. Therefore, more research is required in this field while the pandemic lasts, especially with people with obesity.

**Plain English summary:**

Overall, people with overweight or obesity are more vulnerable to weight stigma than individuals without overweight. In addition, weight stigma refers to discrimination or prejudice based on a person’s weight and relates to several consequences, for instance, poor healthcare treatment and mental health problems. In the COVID-19 outbreak, these weight stigma effects tend to become even more critical because they may be associated with unfavourable COVID-19 outcomes and eating disorder risks. Thus, it is crucial to investigate how weight stigma occurs during the pandemic and its impact on health, mainly for the most affected people.

We investigated 35 studies published between 2019 and 2021 to map and explore how weight stigma was manifested and the related consequences for people with overweight or obesity in the COVID-19 pandemic. Only about a third of them were quantitative or qualitative, limiting the evidence of weight stigma in the COVID-19 context. The available evidence suggests that weight stigma manifests in several settings such as media, healthcare, public campaigns, and is more common in people with excess weight. However, weight discrimination experiences before or during the pandemic were associated with adverse psychological and behavioural consequences across all age groups, regardless of body weight. For some people with obesity, for instance, weight stigma made it difficult to accomplish their activities of daily routine. Nevertheless, it remains unclear whether weight stigma has increased in the pandemic, thus, more studies are required, especially about people with overweight or obesity.

**Supplementary Information:**

The online version contains supplementary material available at 10.1186/s40337-022-00563-4.

## Introduction

The debate about weight stigma and its consequences for people with obesity has gained ground in the scientific community in recent years. In March 2020, experts published the first international consensus to combat weight stigma [1]. Furthermore, with the advent of the COVID-19 pandemic, the media, scientists, and the general public increased focus on body weight [2, 3]. In this sense, the fact that obesity is considered a risk factor for the aggravation of the viral infection and the emergence of the widespread fear of gaining weight due to social distancing measures have become of concern for people living with obesity during the pandemic and reinforced the implications of weight stigma [3–6].

Weight stigma refers to people's devaluation and social depreciation due to excess weight [1], and its multidimensionality encompasses structural and individual forms of discrimination [7]. Structural stigma occurs when institutions as the media and the health sector, for example, issue stigmatizing messages and frame a group negatively [7, 8]. Individual forms of discrimination include person-to-person weight stigma experiences and self-stigma (or internalized weight stigma) [7, 8]. In addition, when high body weight converges with other forms of stigmatization (e.g., socioeconomic status), it can be incorporated into the concept of intersectional stigma [9]. Thus, weight stigma extends to multiple domains such as health services, education, work, family, public policy campaigns, media, and others [1, 10, 11].

In the COVID-19 pandemic, experts suggest increased vulnerability to weight stigma in the healthcare sector and the media [3, 4, 6]. Although these sectors already recognized weight stigma as pervasive in the pre-pandemic environment, it is believed that the increase in media and scientific coverage of obesity and COVID-19 can intensify weight discrimination in healthcare settings [4]. Furthermore, the "quarantine-15" hashtag, which alluded to the fear of gaining weight in the context of social isolation, helped identify the dissemination of stereotyped images of people with high body weight on social media [3, 6].

This information regarding the manifestations of weight stigma in healthcare and the media in the pandemic is remarkably relevant considering that weight-based discrimination is associated with varied consequences. For example, structural stigmas, such as those linked to healthcare settings, are related to behavioural effects such as avoiding or delaying the search for health services [12]. Moreover, weight stigma experiences or internalization are associated with adverse psychological and behavioural outcomes, such as poor mental health [7] and disordered eating [13]. Therefore, in a context of health and economic crisis such as the pandemic, it is necessary to understand how the manifestations of weight stigma can impact health and well-being, especially of the most vulnerable, that is, people with overweight [14].

Experts consider that weight bias in the healthcare settings may be associated with worse outcomes of COVID-19 [4, 5], especially in racial or ethnic minorities and socioeconomically disadvantaged groups affected by other forms of discrimination [5], and that exposure to fat-phobic content in the media could pose a greater risk for worsening eating disorders symptomatology (ED) in times of pandemic [6]. However, a narrative review with articles published in 2020 identified that more studies are required to investigate weight bias and the consequences of weight stigma experienced in the pandemic on health and well-being [15]. Although these results are essential, we believe that an updated review with systematic methodology is necessary to identify which scenarios manifested weight stigma, whether it has increased or not, and its consequences in the context of the COVID-19 outbreak.

Given that the COVID-19 pandemic implies a broad and rapidly developing literature base, a scoping review can provide evidence on the extent, nature, and consequences of weight stigma in that context. Furthermore, scoping reviews are helpful when one has complex and heterogeneous issues [16, 17] and provide an overview of the available scientific literature [18]. Thus, we systematically reviewed the literature published from 2019 to 2021 to explore and map the evidence on weight stigma directed at people with overweight and obesity in the context of the COVID-19 pandemic. We proposed three research questions to meet the objective of this review: (1) How does weight stigma manifest in the COVID-19 pandemic? (2) How can weight stigma affect people with obesity in times of COVID-19? (3) What are the perceptions and experiences of weight stigma during the pandemic in individuals who experience overweight or obesity?

## Method

This scoping review was conducted following the Joanna Briggs Institute methodology [18] and adopted the requirements of the Preferred Reporting Items for Systematic Reviews and Meta-Analyses checklist extension for Scoping Reviews (PRISMA-ScR) [19]. The review protocol was previously registered (https://osf.io/ujcbf).

### Eligibility criteria

The (PCC) framework guided the inclusion of studies in this review. The population (P) of interest was represented by all studies that addressed weight stigma towards people with overweight or obesity; weight stigma was the concept (C) underlying this work, and the Context (C) referred to the COVID-19 pandemic. For the definition of weight stigma in this review, we adopted the proposals by Rubino et al. [1], referring to this phenomenon as the social devaluation and denigration of people because of their excess weight, which can lead to negative attitudes, stereotypes, prejudice, and discrimination. Thus, studies needed to clearly emphasize weight stigma (the presence of the concept, or interchangeable terms, in the study full text). Inclusion criteria consisted of observational, qualitative, or mixed studies and text and opinion evidence. The research excluded in vitro intervention studies involving animals, theses, dissertations, and abstracts.

### Information sources and search strategy

We searched without restrictions in February 2021 and last updated it in August 2021 to identify potentially relevant documents in the following databases: Medline/PubMed, CINAHL, Embase/Elsevier, PsycInfo, Regional Portal BVS/Lilacs, Scopus, and Web of Science, as well as Google Scholar (retained records from the first 15 pages) and OpenGrey (grey literature). Two authors (PFCM, EPM) elaborated the research strategy in three stages. First, an initial search was performed in MEDLINE/Pubmed and PsycInfo. Next, a second search with all keywords and indexed terms was conducted in all databases. Finally, the reference list of all included articles was manually searched to include additional studies. Search terms included “weight stigma” and “COVID-19” and their respective related terms using Boolean operators AND and OR. The final search strategy can be found in an Additional file 1.

### Selection of sources of evidence

After duplicates removal in EndNote®, three authors (PFCM, CMMN, LCL) independently selected studies using the Rayyan software [20]. During the screening, the titles and abstracts of the publications were read, followed by the complete reading and selection of eligible studies. Finally, a fourth reviewer’s (MLPS) decision resolved any discrepancy.

### Data mapping process and data items

The first author (PFCM) extracted the required data from the selected studies by using the Joanna Briggs Institute's System for the Unified Management, Assessment and Review of Information (SUMARI), and two authors (NSS, VAOQ) reviewed the collected information. The extracted variables consisted of the type of study, country of publication, study methodology, the phenomenon of interest (weight stigma and related constructs), subjects (i.e., people with overweight or obesity), context, and relevant findings for the purpose(s) of the review. These variables were recorded as described in the studies or marked as not applicable.

### Synthesis of results

A narrative synthesis reported the results of this review. The collected data were presented in tables or figures. To investigate weight stigma manifestation (question 1), we identified the terms used to refer to the concept, weight stigma dimensions (experienced, self-stigma, structural, intersectional), measurements of the concept (when applicable), and in which domains the phenomenon had occurred (when applicable). In addition, the research included all studies that proposed outcomes or consequences related to the phenomenon to analyse data on the consequences of weight stigma in the pandemic context (question 2), as psychological outcomes measured in primary studies or increased risk of mortality presented in texts and opinion articles. Similarly, to find out about perceptions and experiences of weight stigma in individuals who experienced overweight or obesity during the pandemic (question 3), we considered studies that related directly to the topic, through interviews in qualitative studies, for instance, or discussed the theme in text and opinion documents.

## Results

### Selection and characteristics of sources of evidence

**Fig. 1:**
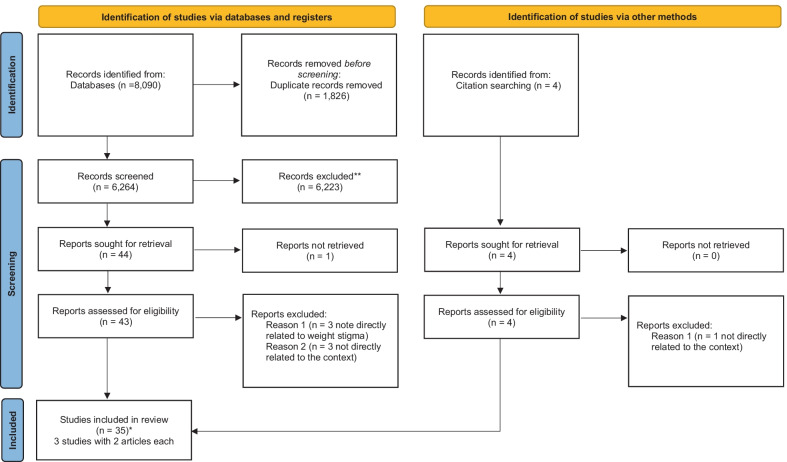
Flow diagram of included studies

A total of 8090 records were retrieved from the databases. After duplicate records had been removed, 6264 studies were screened by title or abstract, and 40 were considered for full text-reading, resulting in 32 original studies [2–6, 15, 21–52] for inclusion (three studies had two published articles each) [26, 32, 38, 43, 48, 49]. Later, the references of the studies included initially were searched, and four additional citations were considered for full text-reading, with three of them fulfilling inclusion criteria [4, 23, 41], totalling 35 included studies for this review. Overall, six unrelated studies to the phenomenon or context of interest were excluded. The selection process of this study is described in the PRISMA flowchart adapted from Page et al. [53] (Fig. 1).

As for the characteristics of the studies, the majority corresponded to text and opinion documents (n = 15) [2–5, 25–27, 29, 31, 33, 36, 37, 41, 42, 50, 52] and narrative reviews (n = 6) [6, 15, 28, 30, 35, 46]. Quantitative (*n* = 6) [24, 26, 32, 38, 39, 43, 47–49] and qualitative research studies (*n* = 8) [21–23, 34, 40, 44, 45, 51] were retained. Of the quantitative studies, three were cross-sectional [24, 39, 47] and three were longitudinal [32, 47–49]. And, among the qualitative studies, seven corresponded to content analysis of texts or images [21–23, 40, 44, 45, 51] and one to people with obesity interviews [34]. (Table 1).

**Table 1:** General characteristics of evidence sources included

Characteristics	No. (%)
**Study type**	
Text and opinion papers	15 (42.8)
*Opinion pieces*	4 (11.4)
*Commentary*	3 (8.5)
*Position statement*	2 (5.7)
*Editorial*	5 (14.2)
*Report*	1 (2.8)
Narrative reviews	6 (17.1)
Quantitative studies	6 (17.1)
*Cohort*	3 (8.5)
*Cross-sectional*	3 (8.5)
Qualitative studies	8 (22.8)
*Interview*	1 (2.8)
*Analysis of media content or public campaign*	6 (17.1)
*Analysis of free-text responses*	1 (2.8)
**Study focus**	35 (100)
Obesity and COVID-19	17 (48.5)
Weight stigma and COVID-19 pandemic	1 (2.8)
Weight stigma in the media during COVID-19 pandemic	2 (5.7)
Eating disorders risk and COVID-19 pandemic	2 (5.7)
Problematization of fatness and COVID-19 pandemic	1 (2.8)
Media/public campaign analysis in the COVID-19 pandemic	6 (17.1)
Weight stigma measured and COVID-19 pandemic	6 (17.1)

**Fig. 2:**
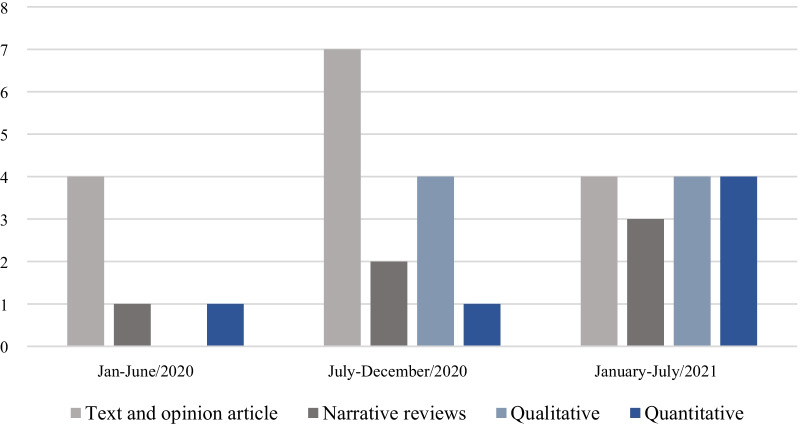
The number of studies published by type of publication throughout the COVID-19 pandemic

Most studies were published in 2020 (*n* = 20) [2–6, 21, 28, 29, 31, 34, 36, 37, 41, 44–48]. As shown in Fig. 2, there were more opinion texts and narrative reviews than quantitative and qualitative studies in the first year of the pandemic. There was great variability related to weight stigma domains addressed by the studies. Thus, it is worth considering the focus of the respective document. Almost half of the studies [4, 5, 25, 27–31, 33, 34, 36, 37, 41, 46, 51] addressed weight stigma when evaluating aspects related to obesity in the pandemic context, and only six [24, 26, 32, 38, 39, 43, 47–49] directly measured variables related to weight stigma (Table 1).

**Fig. 3:**
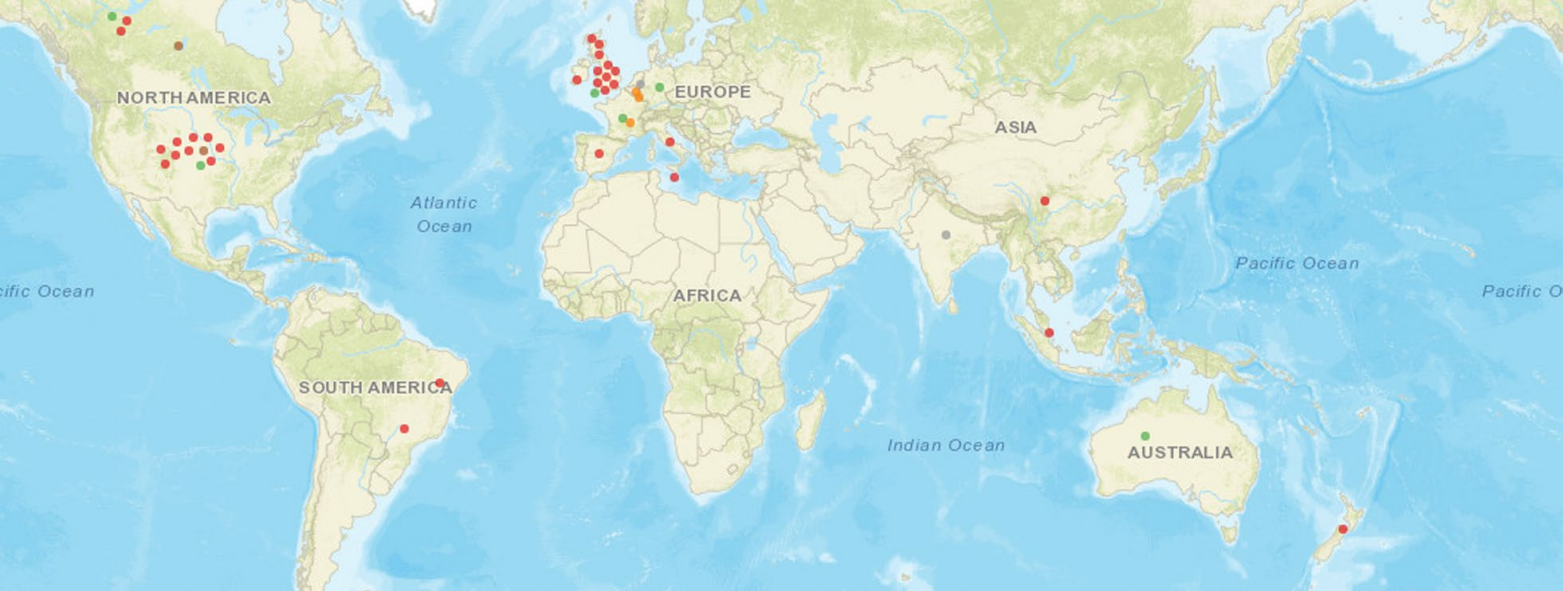
Sources of evidence on weight stigma during the COVID-19 pandemic published by country. Note**:** red dots correspond to different studies (*n* = 31); grey, orange, and brown dots represent three studies and their repetitions stand for the countries of their respective authors; green dots correspond to the samples of a multinational study comprising six countries

Overall, studies were predominantly from Europe (*n* = 19) [2, 4, 22–24, 28–31, 33, 34, 37, 38, 41–43, 46, 47, 54] and North America (*n* = 14) [3, 5, 6, 15, 21, 25, 36, 38–40, 43, 45, 47–49, 51]. One opinion piece [4] and two reviews [6, 30] included authors from different countries. And, one quantitative research used a multinational sample [38, 43]. Figure 3, elaborated via EviAtlas [54], shows a map of study locations and illustrates their concentrations.

### Participants' characteristics

Six quantitative [24, 26, 32, 38, 39, 43, 47–49] and one qualitative study [34] assessed participants’ characteristics. All studies included both genders [24, 26, 32, 34, 38, 39, 43, 47–49] with a sample size of 23 to 13,996. Of these studies, five included a sample of adults (age ≥ 18 years old) from the United States (the US) [38, 43, 47–49], Canada [38, 43], Germany [38, 43], France [38, 43], Australia [38, 43], the United Kingdom (the UK) [24, 38, 43] and Ireland [34]; the other two involved American adolescents (age < 18 years old) [39] and Chinese children (age 7–13 years old) [26, 32]. In addition, two studies with adults included only participants with obesity [24, 34].

### Results from individual evidence sources

The following topics will describe the mapping related to the review questions (Table 2). Additional information is available in an Additional file 2.

**Table 2:** Results mapped according to the three review questions

Type of evidence (No. of studies)	Findings (No. of studies)
**Question 1. Configuration of weight stigma in the pandemic (n = 35)**	
Nomenclature*(35)	Weight stigma (18)
	Obesity stigma (9)
	Weight bias (4)
	Weight discrimination (3)
	Weight bias and stigma (3)
	Others (4)
Cited dimension*(35)	Stigma experienced (pre or during pandemic) (7)
	Self-stigmatization (3)
	Structural weight stigma (25)
	Weight stigmatizing media content (news, social media) (18)
	Weight bias in the healthcare** (13)
	Stigmatizing public health campaigns (5)
	Intersectional stigma (4)
	Not specific / not clear (6)
Measurement* (6)	Experienced/perceived weight stigma (6)
	Single question (3) 3 closed-ended questions (1) 4 closed-ended questions (1) Adapted scale (1)
	Weight stigma internalization (1)
	Scale (1)
	Exposure weight stigma media content (1)
	Single question (1)
Settings/nature* (23)	Media (18)
	Healthcare (13)
	Public campaign (5)
	Public perception of obesity (1)
	Parents and peers (1)
	Online teaching (1)
**Question 2. Consequences/outcomes in the pandemic associated with weight stigma (n = 25)**	
Pre-pandemic/history of weight stigma experiences and pandemic outcomes in quantitative studies* (5)	Decrease in psychological wellbeing indicators in adults (4)
	Greater precautionary behavior to prevent infection in adults (2)
	Association with maladaptive eating in adults (2) ***
	Association with adverse eating behaviours and exercise attitudes in adults (1)
	Predictor of all types of psychological stress in children (1)
Perceived weight stigma during the pandemic and outcomes in quantitative studies (3)	Predictor of all types of psychological stress in children (1)
	Aligned with increased body dissatisfaction during the pandemic in adolescents (1)
Internalized weight stigma measured the pandemic and outcomes in quantitative studies (1)	Association with adverse weight-related health indices in adults (1)
Exposure to weight stigma on social media during the pandemic and outcomes in quantitative studies (1)	Aligned with increased body dissatisfaction during the pandemic in adolescents (1)
Weight stigma and associated consequences in qualitative studies (1)	Decreased ability to engage in obesity treatment during the pandemic because of self-stigmatization in adults (1)
Weight stigma and associated consequences in reviews and text and opinion documents* (18)	Impediment/delay in accessing information/health services in the pandemic (10) as a consequence of the weight stigma suffered in the healthcare (5), previous experiences of weight stigma (3), high stigma/perceived vulnerability in the pandemic (1), concern to be included as a burden to health system(1) and intersection of vulnerabilities in the pandemic (1)
	Worst outcomes for COVID-19 (8) associated with reluctance/delay in seeking health services in the pandemic (5), poorer quality of care for PwO in the pandemic (1), and intersections of vulnerabilities in the pandemic (3)
	The worsening quality of care provided in the pandemic (2) associated with weight bias in health professionals during the pandemic (2) and lack of health equipment/accommodations for PwO (1)
	Risk of illness by COVID-19 associated with rationing of COVID-19 care or resources for fat people (1)
	Deepening health inequities in face of weight stigma (1)
	The relegation of bariatric surgery as an indication of systematic bias and discrimination towards PwO (1)
	Increase ssusceptibility to disordered or maladaptive eating (6) associated with exposure to stigmatizing weight content on social media in the pandemic (2), stigmatization of obesity in public campaigns (1) , PwO stigmatization history (1); children’s bullying in online education (1), previous experiences of weight stigma (1)
**Question 3. Perceptions/experiences of weight stigma in PwO in the pandemic (n = 2)**	
Perceptions/experiences in a qualitative study(1)	Difficulty in getting involved in the treatment of obesity in the pandemic associated with weight self-stigma (1)
Perceptions/experiences in a text and opinion document* (1)	Feeling like are low priority than any other condition as a result of stigma on social media during the pandemic (1)
	Impediment to exercise/buy food in the pandemic associated with stigma or shame (1)

*PwO* people with obesity

*More than one finding may have been identified in a single document

**Evidence of lack of structure or equipment for people with obesity, reluctance to seek help, or weight bias by health professionals

***Engaging in more eating to cope or binge eating

#### Weight stigma manifestations in the pandemic context

All thirty-five studies indicated weight stigma manifestations in the pandemic. Most studies used more than one nomenclature to address the same concept. Nevertheless, the predominant term was weight stigma (*n* = 18) [2, 3, 5, 6, 15, 25–27, 30–39, 43, 44, 47, 52]. Similarly, most documents addressed more than one dimension of the concept, highlighting a higher frequency in the category of structural weight stigma (*n* = 25) [2–6, 15, 21–23, 25, 29–31, 33, 36, 37, 39–42, 44–46, 50, 52], comprising publications about stigmatizing messages disseminated in the media (*n* = 18) [2–4, 6, 15, 21, 23, 27, 30, 31, 35, 37, 39, 40, 42, 44, 45, 52], public health campaigns (*n* = 5) [22, 27, 33, 41, 50] and healthcare (*n* = 13) [1, 2, 4, 5, 15, 25, 29–31, 36, 37, 42, 46] in the pandemic.

Ten studies reported the increase in weight stigma during the pandemic in the media [3, 6, 23, 27, 30, 31, 39], in healthcare [4], on public campaigns [22], expressed as a general vulnerability for people with elevated body weight [42] or related to perceived weight discrimination [39]. Most of these sources of evidence corresponded to expert opinion (*n* = 7). Only two studies observed that weight stigma empirically increased during the pandemic: one qualitative research about the UK’s mass media [23] and one quantitative research with American adolescents [39]. The former found that the British press had created rhetoric around obesity generating more stigma than usual. In the latter, 53% of the teenagers reported increased weight stigma on social media and 12.8% in their interpersonal relationships [39]. More details are described in sections "Media" and "Experienced and internalized weight stigma estimates".

Six studies [24, 26, 32, 38, 39, 43, 47–49] used measures to assess weight stigma: five applied closed-ended questions (one to four items) to analyse weight stigma experiences [24, 38, 43, 47–49] or weight stigma in media content exposure [39]; two studies used scales to assess weight stigma internalization [43] and perceived weight stigma [26, 32]. In addition, a qualitative study [34] observed weight self-stigma through one of the interviews.

Weight stigma in the pandemic has been cited in domains such as the media, healthcare settings, public campaigns, related to perceived experiences and internalization, as mentioned above, in public opinions of people with obesity [51], and interpersonal relationships [25, 39]. Therefore, the following session will feature the evidence on the concept in all respective domains.

##### Media

We classified evidence on weight stigma in the media into two categories: coverage on obesity and COVID-19 and stigmatizing content on social media in the pandemic context.

Two opinion papers [2, 42], one narrative review [15] and three qualitative studies [21, 23, 45] cited the coverage on obesity and COVID-19. Overall, the authors highlighted the narrative of individual responsibility for obesity in the media coverage by journalists [42], broadsheets [23], tabloids [23], television [2], online news media [21, 45], and news stories [2, 15]. The studies suggested that weight stigma in media coverage was either reinforced [2, 21] or intensified [23, 42] in the pandemic. The three qualitative studies identified an implicit anti-fat bias in Brazilian articles published on internet portals [45], Canadian news media [21], and British broadsheets and tabloids [23], but only one analysed pandemic and pre-pandemic periods. Brookes (2021) [23] compared the discourses of articles in British tabloids (*n* = 2000) and broadsheets (*n* = 2000) (published in March-July/2020) to articles on obesity from before the pandemic (August-December/2019). The author concluded that the COVID-19 pandemic produced more stigmatizing discourses than usual, holding people with obesity responsible for their health and the healthcare system's problems [23].

The stigmatizing content on social media during the pandemic was mentioned in seven opinion texts [3, 4, 27, 31, 37, 42, 52], four narrative reviews [6, 15, 30, 35], two qualitative content analysis studies [40, 44] and one quantitative paper [39]. The studies characterized weight stigma on social media as the spread of anti-fat memes and images [3, 15, 31, 40] and messages [6, 37, 42, 44] that mocked people with overweight [27, 30] through jokes about weight gain, sedentary behaviour or overeating during the pandemic [6, 39, 52]. Eight documents mentioned posts on social media referring to "Quarantine 15" [3, 6, 15, 27, 40, 42, 44, 52] characterized by weight-stigmatizing content due to fear or risk of weight gain during the period of COVID-19 social distancing. The two qualitative studies identified anti-fat attitudes on Instagram comments [44] and images [40], which identified posts that disliked higher-weight bodies (46.9%) through the “quarantine-15” hashtag [40]. In the quantitative study (*n* = 452) [32], the majority of adolescents (53%) reported increases in seeing jokes on social media about people eating food because they were stressed (45%) or memes about people gaining weight (37%) during the pandemic. Perceived weight stigma exposure on social media was approximately 10% higher in female adolescents and 20% greater in the girls with higher body weight [32].

##### Healthcare

Eight sources of evidence of text and opinion documents [2, 4, 5, 29, 31, 36, 37, 42] and five narrative reviews [15, 25, 30, 35, 46] mentioned associations to weight stigma in healthcare settings in the COVID-19 pandemic. In addition, at least one dimension of weight stigma, such as experiences of stigma and weight bias, intersections of vulnerabilities during the pandemic, high stigma or perceived vulnerability in the pandemic, or systematic weight bias, was related to consequences for people with obesity in the health sector during the pandemic, such as the impediment or delay in accessing healthcare [2, 4, 5, 15, 27, 29, 31, 36, 37, 46].

##### Public campaigns and public opinion towards people with obesity

Four texts and opinion papers [27, 33, 41, 50] and one qualitative study [22] cited the possibility of further stigmatization of people with obesity through the message of public health campaigns that considered weight loss as a critical strategy to fight COVID-19. Four of these documents referred to the UK public health campaign [22, 33, 41, 50]. In addition, the qualitative study [22] observed personal responsibility discourses directed at people with obesity and argued that the pandemic context itself might have intensified this stigmatization by motivating this new set of policies. Finally, one qualitative study [51] had investigated public opinion towards obesity of Americans living in the United States during the pandemic (May/2020) and observed a predominant narrative about obesity which said that "people are lazy."

##### Interpersonal sources of weight stigma

One quantitative study [39] and one text and opinion document [25] reported potential interpersonal sources of weight stigma. The quantitative study (*n* = 452) showed that part of adolescents perceived an increase in experiencing mistreatment due to body weight from parents and peers in the pandemic context, which was pronounced in people with higher body mass index and females (estimated prevalence in the section below). Moreover, the text and opinion paper [25] suggested that teasing and bullying in online education in the pandemic could also enable children to bully other children with obesity.

##### Experienced and internalized weight stigma estimates

Six studies evaluated measures of experienced weight stigma before or during the pandemic and internalized weight stigma [24, 26, 32, 38, 39, 43, 47–49]. Each study used a different open-ended question or scale and evaluated distinct aspects of the constructs. None of the samples was representative of the wider population including adults, adolescents, and children. In almost all of them [24, 26, 32, 38, 39, 43, 48, 49], weight stigma before or during the pandemic was more prevalent or significantly associated with high body weight; the remaining study did not address this issue [49]. Four of these studies reported the prevalence or mean of weight stigma experienced or internalized in the period of the COVID-19 pandemic [24, 26, 32, 39, 43]. In adults with obesity (*n* = 543), the prevalence of weight stigma experienced during the pandemic was 16.7% [24]. Among adolescents (*n* = 452) [39], 12.8% and 3.8% reported an increase in weight-based mistreatment from parents and peers, respectively, which were almost twice as high in those with overweight [39]. Similarly, in the study with children (*n* = 1357) [26], those with overweight had higher means (*p* < 0.05) of perceived weight stigma scores in the pandemic compared to kids without overweight [26]. However, the children's mean perceived weight stigma scores were higher (*p* < 0.01) than the scores before the pandemic than in the social distancing and post-lockdown period [32]. Only one study investigated internalized weight stigma during the pandemic [43]. They evaluated adults (*n* = 13,996) from six countries enrolled in an international weight management program. This study highlights that the mean internalized weight stigma scores were significantly higher (*p* ≤ 0.001) in the UK, Australia, and France than in Germany, Canada, and the United States. Unfortunately, the authors did not compare the results to values before the pandemic.

#### Weight stigma and associated consequences in the pandemic

Twenty-five included studies reported weight stigma consequences in the pandemic: six quantitative studies [24, 26, 32, 38, 39, 43, 47–49], one qualitative paper [34], six narrative reviews [6, 15, 28, 30, 35, 46] and twelve text and opinion documents [2, 4, 5, 25, 29, 31, 36, 37, 42, 52].

The six quantitative studies [24, 26, 32, 38, 39, 43, 47–49] presented a relation between adverse behavioural and psychological outcomes in the pandemic and weight stigma in adults [24, 38, 43, 47–49], adolescents [39], and children [26, 32]. Additional information from these studies is available in an additional file [see Additional file 2].

Four studies, two cross-sectional and two cohorts, investigated weight stigma consequences in adults. The cross-sectional studies measured the association of history of weight stigma (i.e., had experienced weight stigma until May to July/2020) and psychological and behavioural outcomes in samples composed mostly for people with overweight or obesity [24, 38, 43]. For example, Brown et al. [24] assessed 543 people with obesity from the UK. They observed that participants with a history of weight stigma had lower well-being, more significant depression (*p* < 0.001) and more precautionary behaviours (*p* = 0.010) in the pandemic. The findings of this study accord with more large-scale research [38, 43], in which the authors evaluated 13,996 individuals enrolled in an international weight management program from six countries (the UK, Australia, France, Germany, the US, and Canada). The large-scale research observed that history of weight stigma was also significantly (*p* ≤ 0.001) associated with worsened mental health and some maladaptive behaviours, such as gym avoidance and eating to cope, in the pandemic (May–July/2020) [38]. However, internalized weight stigma was significantly associated with many outcomes, including their effects across countries for poor body image, weight gain in the previous year, less eating and physical activity self-efficacy and lower physical health-related quality of life [43]. The authors also highlighted some differences between countries. For example, France showed slightly weaker effects of weight stigma internalized for some variables, like eating to cope, body image, and stress, when compared to all other countries in the study [43].

Longitudinal studies supported these results. The two cohort studies with adults investigated if pre-pandemic weight stigma experiences could predict psychological distress or behavioural outcomes during pandemic (March and April/2020) [47–49]. These studies sampled people living in the US (*n* = 584–2094) and included assessments before and after the pandemic. Sutin et al. [48, 49] measured responses to the Coronavirus and the trajectory of psychological distress and well-being across the pandemic by BMI category and weight discrimination. Overall, this research observed that pre-pandemic weight stigma experiences were associated with more positive (e.g., engaging in more preventative behaviours) and negative (e.g., less trust in people) responses to the pandemic [48] and influenced the worsening of well-being across the outbreak [49]. The authors also showed that the distress trajectory did not vary by weight discrimination; however, those with pre-pandemic weight stigma experiences were at a twofold increased risk of incident depression [49]. The longitudinal influence of weight stigma on psychological well-being found in this study accords with the research findings of Puhl et al. [47]. These authors observed that pre-pandemic experiences of weight stigma predicted higher levels of depressive symptoms, stress, eating to cope, and an increased likelihood of binge eating (odds ratio = 2.88, *p* < 0.001) [47]. However, nonsignificant associations emerged for depressive symptoms and stress after adjusting for their baseline levels. The authors suggested that the effects of weight stigma were attenuated for distress when considering their pre-pandemic levels [47]. In the two studies [47, 49], the impact of weight stigma persisted regardless of the BMI.

In adolescents, a cross-sectional study with a crosstab analysis showed that increases in weight stigma experienced or in weight stigma exposure on social media by American teenagers (*n* = 542) during the pandemic (until December/2020) were aligned with increases in body dissatisfaction [39]. Furthermore, both weight stigma and body dissatisfaction increase in the pandemic were higher in youth with higher weight (*p* < 0.025) and among females (*p* < 0.001) [39].

Finally, in Chinese children (*n* = 1357), the cross-sectional analyses showed that high levels of perceived weight stigma in the COVID-19 pandemic (March/2020) were significantly associated with greater fear of COVID-19 infection and higher levels of stress, anxiety, and depression regardless of weight status [26]. In the longitudinal evaluation of the same study, (*n* = 489), perceived weight stigma before the pandemic (January/2020) or in subsequent waves (March and June/2020) was associated (*p* < 0.001) with problematic use of social media, depression, anxiety, and stress in all three waves [32]. The association between perceived weight stigma and psychological distress in children was more robust in the pandemic than in the pre-pandemic period [32].

A qualitative study [34] (*n* = 23) pointed out the difficulty of an Irish adult following up on obesity treatment due to self-stigmatization. And, finally, six narrative reviews [6, 15, 28, 30, 35, 46] and twelve text and opinion documents [2, 4, 5, 25, 29, 31, 36, 37, 42, 52] cited negative consequences associated with weight stigma in the pandemic context, in which they highlighted the impediment or delay to information or health services [2, 4, 5, 15, 27, 29, 31, 36, 37, 46], worse COVID-19 outcomes [4, 5, 27, 29–31, 35–37] and increased susceptibility to disordered or maladaptive eating [2, 6, 15, 25, 28, 41, 52]. Some theoretical proposals explained these implications. For example, a position statement elaborated by the European Association for the Study of Obesity (AESO) [29] proposed a conceptual model where past experiences of weight stigma integrated social factors that link obesity with COVID-19 severity. Three opinion articles also suggested the impact of weight stigma associated with other forms of vulnerability in health inequalities and unsatisfactory COVID-19 results [5, 35, 36]. Also, a review [6] pointed out that the increase in fatphobic messaging on media (e.g., “Quarantine 15”) during pandemic integrated the risk factors for worsening symptomatology of individuals with ED. Todisco et al. [52] corroborates this framework and suggest that the weight stigmatizing social media posts in the pandemic may generate common weight-based stereotypes while promoting unrealistic thin ideals and extreme weight-management practices.

#### People with obesity (PwO) perceptions or experiences on weight stigma in the pandemic

Two studies described perceptions or experiences on weight stigma of people with overweight in the pandemic [34, 37]. First, a qualitative study [34] carried out with Irish adult patients (*n* = 23) undergoing multimodal treatment for obesity observed the impact of weight self-stigmatization on the ability to engage in obesity treatment through the one participant’s report. Second, a text and opinion study [37] based on interviews with PwO from the UK showed that participants reported feelings of shame, a perception of being "less of a priority than any other condition" and a reluctance to seek help because of weight stigmatizing comments on social media. In this opinion paper [37], some of the people interviewed associated fear of weight gain during lockdown with stigma or shame, which prevented them from exercising or shopping for food.

## Discussion

### Summary of evidence

The results pointed to four main settings or sources of weight stigma in the COVID-19 pandemic: media (18/35), healthcare (13/35), public campaigns (5/35), and interpersonal relationships (2/35). Weight stigma’s consequences varied according to the study type; quantitative studies (6/35) related weight stigma to behavioural and psychological outcomes in adults (4/6), adolescents (1/6), and children (1/6). Opinion papers and narrative reviews (21/35) suggested delay or impairment in accessing healthcare (10/21), worst COVID-19 outcomes (8/21), maladaptive eating (6/21), and others (3/21). Few studies (2/35) described experiences or perceptions of people with obesity. This work proposes to discuss, firstly, the question on weight stigma manifestations, second, the related consequence, and, thirdly, PwO perceptions and experiences.

Although weight stigma occurs in different sectors during the pandemic, some of these manifestations still need to be tested empirically. For example, although experts have widely mentioned the health sector as a possible source of stigmatization in the pandemic [2, 4, 5, 29, 31, 36, 37, 42], no empirical studies have evaluated this issue. In addition, the scope found in the investigation regarding the different environments that manifested weight stigma in the pandemic partially demonstrates what is already pointed out in studies in the pre-pandemic period, that is, weight stigma is pervasive and manifests itself in multiple domains [1, 14, 55]. Our review also showed conceptual proposals that support the importance of assessing multiple forms of stigma (e.g., weight stigma and mistreatment based on race/ethnicity) [5, 29, 35, 36]. However, the pre-pandemic scenario [56] lacks quantitative data on this issue, indicating a lack of investment in research independent of the outbreak.

What makes empirical evidence even more limited in this context is that only one epidemiologic study assessed weight stigma sources during the COVID-19 pandemic (media and interpersonal relationships) [39]. Besides, this research collected data from an unrepresentative sample of American adolescents, and used single-item assessments that had not previously been validated as instruments to evaluate weight stigma [39]. In any case, higher BMI was associated with weight stigma in the quantitative studies [24, 26, 32, 38, 39, 43, 47–49], which agrees with the literature conducted before the outbreak [14] and suggests the greatest vulnerability of this group. Only one quantitative study with children compared pandemic and pre-pandemic values of weight stigma [26]. The authors suggested that lower perceived weight stigma values during the pandemic may be related to decreased social interactions [26]. Given that the scale used in this research did not assess sources of experiences, we believe that instruments should include them to be more sensitive to understanding how changes in experiences of weight discrimination occurred in the pandemic compared to the previous scenario.

Regarding weight stigma consequences, the empirical evidence available on this subject comes from three cross-sectional papers and three longitudinal studies: the adult studies by Puhl et al. [47], Sutin et al. [48, 49], Lessard et al., Pearl et al. [38, 43] and Brown et al. [24]; the adolescent studies by Lessard and Puhl [39]; and the children's investigation by Chen et al. and Fung et al. [26, 32]. Altogether, the data showed that experiencing weight stigma could lead to unfavourable psychological and behavioural outcomes in the pandemic in all age groups, regardless of body weight. However, some considerations are crucial to interpret these results. First, the research included a small number of studies, all with unrepresentative samples and some partly composed of people who mainly were overweight [24, 38, 43]. Second, although the relationship between weight stigma and worse mental health indicators and maladaptive behaviours in people with or without overweight agrees with previous literature [7, 13, 57], it is impossible to conclude whether this impact is different between the two groups in the pandemic. Third, the consequences for physical or mental health and whether a possible increase in weight stigma can result in worse health outcomes are unclear when stigma comes from different domains (e.g., interpersonal relationships *versus* media) or multiple sources in the outbreak.

Cross-sectional and longitudinal studies supported results on the consequences of weight stigma, particularly in studies with adults. In addition, previous studies demonstrate the relationship between weight stigma (experienced and internalized) with emotional distress and worse body image and symptoms of eating disorder (ED) [58, 59]. Therefore, based on the data collected during this review, we suggest that this may be a pathway to increase the risk of eating disorders at the present. Furthermore, differences in the frequency and impact of weight stigma across countries may indicate that cultural issues mediate these relationships [38, 43], and one should proceed with caution when comparing results. Furthermore, we believe that a more robust statistical analysis for repeated data can help to understand better the effect of weight stigma on distress throughout the pandemic. The increased weight stigma and body dissatisfaction in adolescents during the pandemic [39] indicate significant findings; however, the theme also demands more robust statistical analyses to confirm these results and their weight status and gender consequences. In children, the influence of weight discrimination on mental health appears to have been exacerbated throughout the pandemic [32]. Although a meta-analysis suggests that age is not a moderator of the effect of weight stigma on emotional distress in the pre-pandemic setting [7], children may be particularly susceptible to the adverse effects of the pandemic on mental health as they have limited coping strategies [60]. Therefore, weight stigma may be amplifying worse mental health outcomes in an already vulnerable group during the outbreak.

When these findings from quantitative studies are considered together with well demonstrated diminished mental health [7] and disordered eating association [13] in people who have suffered weight stigma, they support the importance of considering weight stigma in mental health and eating disorder (ED) risks in times of great stress, such as the pandemic. Furthermore, it is also essential to consider the susceptibility of gender and weight differences to increased exposure to weight stigma content and body dissatisfaction, which can be crucial in identifying vulnerable groups to ED in the current scenario.

Furthermore, several papers pointed out the implications of weight stigma during the pandemic in worse healthcare quality [4, 29, 30, 36, 37, 42], mainly for people with obesity. Even though this evidence was taken from opinion papers, it is worth recognizing and evaluating whether weight stigma in the context of health crisis could imply worse outcomes related to COVID-19 and health disparities. Finally, despite obesity being a risk factor for COVID-19, studies have shown that media coverage and public policies have used this premise to reinforce or intensify anti-fat attitudes through weighty stigmatizing narratives and images [2, 22, 23]. However, no included study evaluated the influence of media coverage and public policies launched in the pandemic on the health and well-being of people with obesity. We believe that identifying groups vulnerable to COVID-19 is vital to establishing effective protective measures. Weight stigma can pose additional questions to people living with obesity, and therefore, its consequences need to be appropriately investigated.

According to studies based on interviews with people with obesity included in this review [34, 37], the findings may also reflect possible challenges faced in the pandemic due to weight stigma, such as difficulty or reluctance to perform activities of daily living. Similarly, previous qualitative research published before the pandemic shows that weight stigma (experiences or self-stigmatization) contributed to depressive feelings [61] and avoidance behaviour towards situations in which individuals perceive that they could be stigmatized [62]. Although it is possible to consider that subjectivity does not just consist of the relationship with the body, it is necessary to assume that weight stigma is a constitutive part of people's personal experience. In this respect, this review did not identify any study that directly investigated qualitatively what people with obesity feel or perceive about weight stigma during the pandemic.

### Knowledge gaps and proposals for future studies

The results of this review indicated the scarcity of primary studies that identified the frequency and impact of individual (experienced weight stigma or self-stigmatization), structural or intersectional dimensions of weight stigma assessed through scales, self-reports, interviews, focus groups, or reports during the pandemic. Thus, representing critical gaps in the evidence. Furthermore, interpersonal sources of weight stigma were measured only by one investigation with adolescents and mentioned in online teaching in a single opinion paper. Home confinement in the pandemic impacts relationships [63, 64] and imposes novel dynamics (such as the new norm of increased telecommunication and distance learning) [65]. So, there is a need to investigate how people express weight stigma facing these matters. Identifying weight discrimination in different settings is crucial to designing effective interventions and politics.

No empirical study investigated extreme or unhealthy weight control behaviours regarding weight stigma consequences on the COVID-19 outbreak. Nevertheless, further research is required to better assess the fatphobic dynamics in the care of people with obesity during the pandemic since their negative impact on health and well-being. Moreover, fat activism has combatted normative beliefs that contribute to the pervasiveness of weight bias [66] and, therefore, needs scientific attention on the current scenario.

This lack of evidence is an indication that research on weight stigma in the pandemic needs more attention and more resources, as highlighted above [15]. Furthermore, it is crucial to understand how weight stigma occurs in the outbreak and its consequences to map its extent and nature. Therefore, clarifying research areas for effective implementation of strategies is particularly relevant in this pandemic context because weight stigma may be an additional factor to influence unfavourable health outcomes.

### Strengths and limitations

According to the authors ' knowledge, this work is the first comprehensive scoping review to map and systematically explore weight stigma in the COVID-19 pandemic context. It is an important issue, though, as stigmatization has negative implications for weight-related health correlations and behaviours [7, 55, 57, 67, 68]. However, this scoping review has some limitations primarily because the availability of evidence limits it. We found basically opinion papers (specifically for the intersectional domain and healthcare implications), so the phenomenon was mainly described from researchers' and experts' perspectives, making it difficult to understand to what extent those data could extrapolate to the manifestations of the phenomenon in the pandemic. Furthermore, although there were studies from many countries, they may not apply to all demographic groups. There was a predominance of publications from European countries and the United States, so that it would require a better investigation on weight stigma characterization in other scenarios. Nevertheless, in literature, terms such as prejudice, discrimination, bias, and stigma are commonly used interchangeably within the concept proposed in this review, which may have hindered a more accurate understanding of the phenomenon's dimensions and its respective effects in the pandemic. Finally, we searched for sources of evidence for weight stigma during the COVID-19 outbreak at the end of 2021 with a range of time equivalent to more than a year of the pandemic, but the majority of the quantitative studies found in the present review collected data from March to July 2020, which restricts the understanding of the effects of weight stigma to the initial months of the pandemic.

## Conclusion

In conclusion, weight stigma definitely manifested in the media, public campaigns, and interpersonal relationships during the pandemic. Nevertheless, the evidence of increased weight stigma in the COVID-19 outbreak is limited. Although weight stigma consequences are associated with implications for psychological well-being across all age groups regardless of body weight, people with high body weight were more likely to experience weight stigma before and during pandemic than those without overweight. The scarcity of studies measuring weight stigma in the pandemic context supports the need for high-quality research to identify and confirm weight stigma characterization. Besides, it reinforces the need to understand weight discrimination magnitude in different global contexts and its impacts on people with overweight or obesity, thereby contributing to the development of effective and inclusive public health policies and eating disorders prevention.

## Supplementary Information

**Additional file 1**. Pubmed/MEDLINE Search Strategy. Strategy for PUBMED (Medline) database: conducted August 2021.

**Additional file 2**. Summary of findings

## Data Availability

The datasets used and/or analysed during the current study are available from the corresponding author on reasonable request.

## Abbreviations

ED: Eating disorders
PwO: People with obesity
BMI: Body mass index
US: United States
UK: United Kingdom
