# Supplementary material for: Weight stigma in the COVID-19 pandemic: a scoping review
Source: J Eat Disord. 2022 Mar 26;10:44. doi: 10.1186/s40337-022-00563-4 (PMC8961493; doi:10.1186/s40337-022-00563-4)
Supplement: Supplementary file 1 — Additional file 1. Pubmed/MEDLINE Search Strategy. Strategy for PUBMED (Medline) database: conducted August 2021. [file 40337_2022_563_MOESM1_ESM.pdf]

Additional file 1. Pubmed/MEDLINE Search Strategy

Strategy for PUBMED (Medline) database: conducted August 2021.

(A similar strategy was used in other databases).

Search	Query	Records retrieved
#1	"COVID-19"[Mesh] OR (COVID 19) OR (COVID-19) OR (2019-nCoV) OR (nCoV) OR (Covid19) OR (SARS-CoV) OR (SARSCov2 or ncov*) OR (SARSCov2) OR (2019 coronavirus*) OR (2019 corona virus*) OR (Coronavirus (COVID-19)) OR (2019 novel coronavirus disease) OR (COVID-19 pandemic) OR (COVID-19 virus infection) OR (coronavirus disease-19) OR (2019 novel coronavirus infection) OR (2019-nCoV infection) OR (coronavirus disease 2019) OR (2019-nCoV disease) OR (COVID-19 virus disease) OR (coronavirus pandemic)	172,014
#2	"Weight Prejudice"[Mesh] OR (weight prejudice) OR (Obesity Bias) OR (weight stigma*) OR (discriminat*)	286,638
#3	#1 AND #2	1266
