# Supplementary material for: Weight stigma in the COVID-19 pandemic: a scoping review
Source: J Eat Disord. 2022 Mar 26;10:44. doi: 10.1186/s40337-022-00563-4 (PMC8961493; doi:10.1186/s40337-022-00563-4)
Supplement: Supplementary file 2 — Additional file 2. Summary of findings [file 40337_2022_563_MOESM2_ESM.pdf]

Additional file 2. Summary of findings

Authors, year, location	Study type	Methodology	Relevant findings to the purpose(s) of the review
**TEXT AND OPINION AND REVIEWS**			
Flint S, 2020, UK	Opinion Article	N/A: text and opinion study	• The media blames and responsibilizes individuals for obesity and uses stigmatizing images and language during the COVID-19 pandemic.<br>• The news has constantly conveyed a tone of stigma when informing about the seriousness of COVID-19 and obesity.<br>• Reports give account of people who are avoiding healthcare because they do not wish to "burden" the health system.<br>• Stigmatizing attitudes and internalized stigma can be developed through the influence of media coverage and lead to harmful behaviour such as binge eating.<br>• Experiences of weight stigma and discrimination during the COVID-19 pandemic can be a direct or indirect result of media portrayals.
Pausé C, Parker G, Gray L, 2021, New Zealand	Opinion Article	N/A: text and opinion study	• The vulnerability to stigma, prejudice and discrimination of people with higher body weight to COVID-19 will increase with exposure to the problematization of fatness.<br>• The problematization of fatness has been amplified by its use by the government, public health officials, and journalists. Anti-fat attitudes were also expressed on social media platforms and COVID-19's proposals for rationing care and resources for people with higher body weight.
Miller G, 2021, UK	Opinion Article	N/A: text and opinion study	• People with obesity can remain stigmatized if there is the perception of a link between COVID-19 and 'lifestyle' factors, as in the message conveyed in the UK's government campaign.<br>• Stigma and shame will increase if messages about weight loss targeting people with obesity remain unchallenged.
Pearl RL, 2020, US	Perspectives	N/A: text and opinion study	• Weight-stigmatizing content is seen with the increase in social media posts referring to "quarantine-15".<br>• Social media posts that stigmatize obesity are harmful to people across the weight spectrum but may be more detrimental to patients actively trying to control their weight.
Le Brocq S, Clare K, Bryant M, Roberts K, Tahrani AA, 2020, UK	Comment	N/A: text and opinion study	• PwO realized stigma in the pandemic context related to comments on social media and the media in general.<br>• Many PwO reported that such comments led to feelings of shame, a perception of being "less of a priority than any other condition," and a reluctance to seek help. Some PwO also related fear of weight gain during lockdown to stigma or shame, which prevented them from exercising or shopping for food.<br>• Stigma towards PwO can either be conscious or unconscious and be delivered from multiple sources, including health professionals. One consequence can be avoidance of healthcare, probably worsening COVID-19 outcomes.
Townsend MJ, Kyle TK, Stanford FC, 2020, US	Commentary	N/A: text and opinion study	• Weight bias exerts an overall powerful effect in medical and public settings and is likely to impact COVID-19 epidemiology.<br>• As a result of an emphasis on obesity as a risk factor for COVID-19 by government guidance and news' sources, the heightened stigma or perceived personal vulnerability people with obesity to delay seeking health care.<br>• Healthcare avoidance may act synergistically with any underlying biological susceptibility to critical illness and mortality among PwO. The harmful effect of weight bias falls disproportionately on the minority and socioeconomically disadvantaged groups most affected by obesity, structural barriers to health, racism, and other forms of discrimination, thus exacerbating the inequities now evident in cases of COVID-19 and mortality.
Hill MA, Sowers JR, Mantzoros CS, 2020, US	Commentary	N/A: text and opinion study	• Weight stigma can be reflected in the reluctance by patients to seek healthcare and poor care deliverance due to a negative stereotype that health professionals may unwittingly apply to PwO<br>• These weight stigma situations add to the mechanistic and social interactions that occur between obesity and unsatisfactory COVID-19 outcomes.
Dicker et al, 2020, UK	Position Statement	N/A: text and opinion study	• The intersection of vulnerabilities such as socioeconomic status, ethnicity, language, gender, immigration status, and previous weight stigma in healthcare can prevent PwO from accessing COVID-19 information and healthcare services.<br>• Weight bias and obesity stigma may affect the quality and access to care for COVID-19. Biased beliefs and attitudes can also influence clinical judgment and behaviours. For example, health professionals may conduct less PwO monitoring. The relegation of bariatric surgery to the bottom of the surgical lists in several countries may indicate inequity, systematic bias, and discrimination towards PwO.<br>• Weight bias and obesity stigma are social factors that relate obesity to the severity of COVID-19.

Frühbeck et al. 2020, UK	Position Statement	N/A: text and opinion study	• The increased of fat shamming memes' on social media may perpetuated misconceptions regarding PwO in the pandemic.<br>• Healthcare weight stigma experiences can cause PwO to avoid or delay contacting health care professionals during the pandemic, resulting in more severe outcomes of COVID-19.
Bhasker AG, Greve JW. 2020, India; the Netherlands	Letter to the Editor	N/A: text and opinion study	• COVID-19 patients suffering from obesity are experiencing increased levels of bias.<br>• Bias against individuals suffering from obesity includes stereotyping them as lazy, less active, and having less willpower, which is further reinforced by the weight-based memes and stigmatizing content present on social media in the pandemic.<br>• Weight stigmatization may also happen when patients suffering from obesity seek health services for COVID-19<br>• Due to the hypothesis of obesity as an aggravating factor for COVID-19, dissuading from bariatric surgery also adds to the stigma against patients with obesity.<br>• Weight stigma can impact the quality of care provided and lead to unfavourable COVID-19 results in PwO.
Chua, MWJ, 2021, Cingapura	Letter to the Editor	N/A: text and opinion study	• Weight stigma has worsened during the pandemic due to several factors such as increased stress, negative affect, and rumination.<br>• Over-emphasizing or attributing worse COVID-19 outcomes to obesity alone leads to further stigmatization and discrimination against people with obesity. Public health campaigns that consider weight loss as a key strategy to fight COVID-19 can worsen the problem.<br>• Mass and social media can also perpetuate weight stigma through posts such as quarantine-15 that make fun of overeating and people with obesity.<br>• Previous weight stigma experienced in healthcare settings can lead to late diagnosis, worse prognosis, and increased viral transmission in people with obesity during the outbreak.
Todisco P, Donini LM. 2020, Italy	Editorial	N/A: text and opinion study	• Social media posts which warned against the risk of weight gain during social distancing due to the vulnerability to overeating and sedentary behaviour may have acted as a form of weight stigma.<br>• Most of these posts, the so-called "quarantine-15", have directly or indirectly generated common stereotypes suggesting that people with obesity are lazy, shabby, and without self-control, besides promoting unrealistic thin ideals and extreme weight-management practices.
The Lancet Gastroenterology & Hepatology, 2021, UK	Editorial	N/A: text and opinion study	• A concern observed in the pandemic period is that the United Kingdom's healthy eating and weight loss campaign presented as a way to "beat the Coronavirus and protect the National Health System," might reflect stigmatizing weight perspectives with an emphasis on personal responsibility
Nature Reviews Endocrinology, 2020, UK	Editorial	N/A: text and opinion study	• The language used in the UK's Government's political campaign published in July 2020 to combat obesity could result in people with obesity being blamed for problems in the health system and in individuals infected by the Coronavirus being likely to feel shame and guilt.<br>• This stigmatization of obesity can also encourage crash diets and an unhealthy relationship with food, which can be detrimental to long-term physical and mental health.
Browne et al., 2021, US	Report	N/A: text and opinion study	• In the pandemic, teasing and bullying in online education can become a means by which children bully other children with obesity, which can be associated with maladaptive eating, stress, anxiety and depression.<br>• Weight stigma internalization also may lead to disordered eating and unhealthy weight.<br>• Prior negative healthcare experiences can also impact an open and trustful relationship with health professionals.
Cooper et al., 2020, US, Canada	Review	Brief review of the pertinent literature related to the risk of EDs in the context of COVID-19	• Fatphobic messages surrounding diet and exercise in the media during the pandemic increased substantially. For example, the media raised the fear of weight gain by alluding to the possibility of gaining weight in "Quarantine 15".<br>• The increase in these messages is considered a specific risk factor for eating disorders in light of the COVID-19 pandemic.
Petrova et al., 2020, Spain	Review	Summary of the scientific evidence available on the role of obesity in COVID-19.	• The stigma of obesity may grow if the influence of obesity in the prognosis of COVID-19 and the transmission of the virus be confirmed and if special preventive measures are adopted for this population.<br>• Previous weight stigma experiences in the healthcare system may make patients with obesity avoid medical appointments until their condition becomes more severe.
Cuschieri S, Grech S, 2020, Malta	Narrative Review	A literature search using 'COVID-19', 'Obesity' and 'Abdominal Obesity' as keywords was performed through Pubmed and Google. Also, international organizations' websites were searched, including WHO, CDC and EASO.	• Individuals who are already stigmatized and have high rates of depression may suffer greater psychological impact with measures of distancing and self-isolation.<br>• There is a greater chance that PwO consumes processed and canned foods instead of fresh foods to avoid going to the supermarket.

Dohet F, Loap S, Menzel A, Iddir M, Dadoun F, Bohn T, Samouda H, 2021, Luxembourg; France; Belgium	Review	Addressing aspects of obesity and COVID-19.	• The increase in PwO teasing and bullying on social media results from public anxiety about possible weight gain related to a pronounced sedentary lifestyle and fear of overeating in the pandemic.<br>• Weight bias by healthcare professionals may be exacerbated during the COVID-19 pandemic due to the unique needs of patients with obesity.<br>• Weight bias and stigmatization in healthcare settings can delay the search for the treatment of obesity and COVID-19, which might lead to a poor prognosis and a probable admission of the patient to an intensive care unit.
Pearl RL, Schulte EM, 2021, US	Review	Selected representative peer-reviewed studies by searching scholarly databases (e.g., PubMed) for articles published in 2020 that included terms such as "COVID-19," "obesity," "eating," "physical activity," "lifestyle change," "weight bias, or "weight stigma."	• Quarantine-15 is a term used in the media to allude to the fear of weight gain in the pandemic and characterized by weight stigmatizing images.<br>• There is a lack of studies to assess the impact of media coverage and public health campaigns (criticized for conveying messages of stigma and blame, for example, individual accountability and use of stereotyped images of individuals with higher weight) on prejudice and stigma.<br>• In healthcare settings, attention should be paid to potential patients prevented from COVID-19-related medical care due to stigma and discrimination.<br>• Although some evidence suggests greater vulnerability to binge eating and mental health problems during the pandemic among those who have suffered weight prejudice in the past, no study to date has evaluated experiences or internalization of weight prejudice and stigma since the beginning of the pandemic.
Gutin I, 2021, US	Review	The purpose of this review is to discuss ontological issues in how BMI is thought and used.	• Social beliefs about worse health status among people with higher BMIs can exacerbate the risk of disease in PwO by increasing propensity for unhealthy behaviour and avoidance of medical care. These beliefs are reflected in the health professionals' assumptions about PwO and emerging pandemic media narratives about who is to blame.<br>• It is crucial to consider the ramifications of how the combination of obesity and COVID- 19 perpetuates a narrative of individual culpability and social burden and that might remain after COVID-19 is no longer a threat.
**QUANTITATIVE STUDIES**			
Brown A, Flint SW, Kalea A, O'Kane M, Willams S, Batterham RL, 2021, UK	Cross-sectional	Study with online data collection during the pandemic period (May to July/2020) with adults with obesity residing in UK	• 16.7% of the sample reported felt stigmatized since the beginning of the pandemic.<br>• 37.4% of the sample felt stigmatized before the pandemic.<br>• Higher BMI increased the chances of having stigma before and during the first COVID-19 lockdown.<br>• Participants who experienced stigma before or during the pandemic take more risk-mitigating actions ($p = 0{,}01$), had lower well-being ($p<0.001$), greater depression ($p<0.001$), higher BMI ($p<0.001$), and were younger ($p<0.001$).
Lessard LM,Puhl RM, Himmelstein MS, Pearl, RL, Foster GD, 2021, Australia, Canada, France, Germany, UK, and US[a]	Cross-sectional	Study with online data collection during the pandemic period (May to July/2020) with adults enrolled in an international weight-control program (Weight Watchers) from six countries (n = 13,996)	• 57.9% of the sample reported history of weight stigma.<br>• History of weight stigma were higher in Canada (61.3%), US (59.6%), UK (58%), than in Australia (56.1%), France (55.6%) and Germany (55.6%).<br>• Participants who had experienced weight stigma engaging in more eating to cope ($\beta = 0.20$, $P < 0.001$), gym avoidance ($\beta = 0.18$, $P < 0.001$), self-monitoring of weight, eating, and physical activity ($\beta = 0.10$, $P < 0.001$), and higher levels of stress ($\beta = 0.15$, $P < 0.001$), as well as reduced eating self-efficacy ($\beta = -0.12$, $P < 0.001$) and exercise self-efficacy ($\beta = -0.07$, $P < 0.001$).<br>• Weight stigma was not negatively associated with exercise self-efficacy in France.
Pearl RL, Puhl RM, Lessard LM, Himmelstein MS, Foster GD, 2021, Australia, Canada, France, Germany, UK, and US[a]	Cross-sectional	Study with online data collection during the pandemic period (May to July/2020) with adults enrolled in an international weight-control program (Weight Watchers) from six countries (n = 13,996)	• Participants from the UK (4.61), Australia (4.46), and France (4.41) had higher internalized stigma scores than (4.18), Canada (4.17), and the US (4.09).<br>• Higher internalization was associated with higher BMI, younger age, and younger age of overweight onset<br>• In all countries, greater internalized weight stigma was associated ($p \leq 0.001$), after controlling for sociodemographic characteristics and experiences of weight stigma, less eating self-efficacy (β = -0.26 to -0.43), less physical activity self-efficacy (β = -0.21 to -0.34) , more eat to cope (β =0.53 to 0.37) , greater gym avoidance (β = -0.36 to -0.42) , poorer body image eating (β = -0.60 to -0.67), lower mental (β = -0.37 to -0.44) and physical health (β = -0.10 to -0.21; $p <0.01 - \leq 0.001$) and greater perceived stress eating (β = 0.39 to 0.50).<br>• In Canada, France, and the UK, who reported weight stigma experiences had greater weight loss.<br>• France showed slightly weaker effects of weight stigma internalized for some variables, like eating to cope, body image, and stress.
Puhl RM, Lessard LM, Larson N, Eisenberg ME, Neumark-	Longitudinal	Study with online collection before (2018) and at the beginning of the COVID-19 pandemic with young adults from Minneapolis/USA (n=584).	• 43% of the sample reported pre-pandemic weight stigma experiences.<br>• Pre-pandemic weight stigma experiences predict higher levels of depressive symptoms ($\beta=0.15$, $p < 0.001$), stress ($\beta =0.15$, $p = 0.001$), eating to cope ($\beta = 0.16$, $p < 0.001$), and an increase in binge eating (odds ratio = 2.88, $p<0.001$) among young adults during the COVID-19 pandemic, but not related to physical activity.

Stzainer D. 2020, USA			• Weight stigma effects persisted for all variables after controlling for pre-COVID-19 BMI.<br>• After controlling for the outcome variables at baseline (except eating to cope, which was not assessed at baseline), weight stigma effects were retained only for binge eating (odds ratio = 2.41, p = .002).<br>• Were no significant interaction based on gender or data collection time (during or after stay-at-home orders).
Sutin AR, Robinson E, Daly M, Gerend MA, Stephan Y, Luchetti M, et al. 2020, US[b]	Longitudinal	Study with data collected online before (February/2020) and during the pandemic (March/2020) of adults living in the USA (n=2,094).	• 14.5% of the sample reported pre-pandemic weight stigma discrimination.<br>• Pre-pandemic weight stigma discrimination were 8.7% in people without obesity and 29.5% in PwO.<br>• Weight stigmatizing experience was associated with greater concern about the virus (p=0.001), less trust in institutions to manage the pandemic (p=0.000), engagement in greater precautionary behaviour to prevent infection (p=0.008), greater perception of decline in affective relationships (p=0.037).<br>• There was no relation between BMI and any of the coronavirus responses.
Sutin AR, Stephan Y, Luchetti M, Aschwanden D, Strickhouse JE, et al. 2020, US[b]	Longitudinal	Study with online data collection in early February, mid-March, and late April 2020 from adults living in the US (n=1,590).	• 11.9% of the sample reported pre-pandemic weight stigma discrimination<br>• Pre-pandemic weight discrimination, rather than BMI, was associated with a twice as high risk of incident depression (p = 0.006), declines in life purpose (p <0.001), and life satisfaction (p<0.001) during the pandemic.<br>• The trajectory of distress did not vary by weight discrimination.<br>• Across all analyses, there was no interaction between BMI and weight discrimination.
Lessard LM, Puhl RM, 2021, US	Cross-sectional	Cross-sectional study with online data collection during the pandemic period (August to December/2020) with adolescents from the US predominantly from the Northeast region (n = 452)	• 53% of the adolescents reported greater exposure to at least one form of weight stigmatizing social media content (stress eating jokes or to weight gain memes).<br>• 45% and 37% of the adolescents reported exposure to stress eating jokes and weight gain memes, respectively.<br>• Exposure to stress eating jokes and weight gain memes was pronounced in female adolescents (55% and 41%) and in adolescents with higher body weight (66% and 48%).<br>• 12.8% and 11.1% of the adolescents noticed an increase in hurtful comments and teasing about their weight by parents during the pandemic, respectively.<br>• 3.8% and 3.4% of the adolescents noticed an increase in hurtful comments and teasing about their weight by peers, respectively.<br>• People with higher BMI had a greater perception of the increase in hurtful comments and teasing about their weight by parents (22.7% and 15.3%) and peers (7.1% and 7.1%).<br>• The level of perception about the increase in hurtful comments and teasing about their weight by parents was even higher in female girls with higher weight (36% and 24%).<br>• Adolescents' exposure to weight stigma and experiences during the pandemic was also in line with the increase in body dissatisfaction over the same period.
Chen C, Chen I, O'Brien KS, Latner JD, Lin C, 2021, China[c]	Cross-sectional	Study with online data collection during the pandemic (March 2020) with primary school children in Sichuan, China (n=1,357).	• Perceived weight stigma during the pandemic was significantly higher in schoolchildren with overweight.<br>• High levels of weight stigma were associated with fear of COVID-19 infection (p<0.001), stress (p<0.001), anxiety (p<0.001), and depression (p<0.001).
Fung XCC, Siu AMH, Potenza MN, O'Brien KS, Latner JD, Chen C, Chen I, Lub C, 2021, China[c]	Longitudinal	Three-wave longitudinal study conducted pre, during, and post-COVID-19 lockdown (January/March/June / 2020) with primary school children in Sichuan, China (n=489).	• Perceived weight stigma before the outbreak was significantly higher than in the two subsequent waves.<br>• Problematic smartphone use, problematic social media, perceived weight stigma and psychological distress were positively correlated with each other in all three waves.<br>• Problematic social media use was increased associated with perceived weight stigma across the three waves in all three models (depression, anxiety and stress).<br>• Although perceived weigh stigma was higher before the pandemic compared to subsequent waves, it was associated, across the three waves, with depression (before: $\beta = 0.317$; during: $\beta = 0.449$; after: $\beta = 0.519$), anxiety (before: $\beta = 0.308$; during: $\beta = 0.388$; post: $\beta = 0.493$) and stress (before: $\beta = 0.357$; during: $\beta = 0.433$; post: $\beta = 0.447$).
**QUALITATIVE STUDIES**			
Grannell A, le Roux CW, McGillicuddy D. 2020, Ireland	Cross-sectional	Qualitative study with a phenomenological approach with semi-structured interviews with obese people undergoing clinical treatment in Ireland (n = 23) during the pandemic (April-May/2020)	• One patient reported the limiting impact of self-stigmatization on his ability to engage in obesity treatment.
Pedrosa VA, Martins PC, Penaforte FR, 2020, Brazil	Thematic analysis	Qualitative study with thematic analysis of comments centered on body exposure of profiles of Brazilian fitness influencers on Instagram (n = 10) published in the	• The reinforcement of the social stigma of obesity was observed in the narratives referring to the great concern with weight gain, associated with changes in eating behaviour and lack of physical activity generated by the quarantine, as well as in the exaltation of thinness linked to the ideas that it is something easy to be achieved and dependent only on individual effort.<br>• The concerns with weight gain and an "ideal" body shape seemed to gain priority in the context of the severe health crisis experienced.

		first month of social distancing in Brazil (March-April/2020)	
Silva, LP, 2020, Brazil	Thematic analysis	Qualitative study with thematic analysis of Brazilian articles published on internet portals (March-May/2020) (n = 25) with the centrality of the theme on food and/or weight loss/gain in the context of the pandemic.	• The author suggests that weight stigmatization was reinforced for articles that oversimplify the weight gain process.
Thomas DM, Siegel B, Baller D, Lindquist J, Cready G, Zervios JT, Nadglowski JF, Kyle TK, 2020, US	Neural language processing	Qualitative study with neural language processing (NPL) of free-text responses (n = 1070) to the question to identify perceptions about obesity, people with obesity, and weight bias of 1114 adults living in the US during the pandemic (May/2020).	• Sentiment analysis showed that emotions of disgust and fear were more commonly associated with Americans' perceptions of obesity than emotions of confidence and joy.<br>• The bigram and trigram charts revealed narratives such as "people are lazy" as a prevalent perception of what the American public thinks about people with obesity.
Lucibello KM, Vani MF, Koulanova A, deJonge ML, Ashdown-Franks G, Sabistona CM, 2021, Canada	Content analysis	Content analysis (n = 668) of posts posted to Instagram (June 2020) with a hashtag #quarantine15.	• The results showed that almost half (46.9%) of the posts dislike towards higher-weight bodies and about a third were considered objectifying (34.4%).<br>• A high proportion of posts perpetuated weight control through diet (51.5%) and physical activity (27.5%).
Bessey M, Brady J, 2021, Canada	Discourse analysis	A qualitative study with discourse analysis of articles (n = 114) from the Canadian news media with the largest circulation (n = 20) published during the pandemic period (March - October 2020) that contained the words "COVID-19" and "obesity".	• Canadian media coverage of COVID-19 and “obesity” frames fatness in highly medicalized, moralized, and individualistic terms that construct health as a matter of moral obligation and personal control.<br>• Anti-fat logics about obesity risk and death discursively problematize fat bodies not only as predisposed to COVID-19, but also by implication, as irresponsible vectors of disease that threaten to overwhelm the healthcare system and that put the community at risk.<br>• Framing obesity as a medical and moral problem in Canadian media coverage of “obesity” and COVID-19 enacts the stigmatization of PwO, which may be seen, further on, as a justification for the mistreatment of PwO and anti-fat-bias.
Brookes G, 2021, UK	Discourse analysis	A qualitative study with identification of discourses through keyword analysis (corpus linguistic technique) of newspaper articles in British broadsheet (n = 2000) and tabloids (n = 2000) that mentioned COVID-19 and obesity published between 1st March a 31st July 2020.	• British coverage of obesity and COVID-19 by broadsheets and tabloids’ analyses has produced a series of discourses around obesity that are more stigmatizing than usual. People with obesity were described in especially fatalistic terms and taken responsible for problems in the country's health system (prominently by the tabloids).
Brookes G, 2021, UK	Discourse analysis	Discourse analysis (CDC approach) from UK policy document "Tackling Obesity: Empowering Adults and Children to Live Healthier Lives" published online on July 27 on the website of the Department of Health and Social Care (2020).	• It was observed that weight stigma could have been intensified by discourses of personal responsibility directed at people with obesity (responsibility for their health, the health of other people, and the health of the NHS), and also by more specific representations such as militaristic metaphors that frame obesity as a "war" or weight loss as a "fight." It is argued that the pandemic context itself may have intensified this stigmatization by motivating this new set of policies, which contributes to exacerbating the physical and emotional turmoil caused by weight stigma and shame.

[a, b, c] Repeated letters represent different articles published from the same study.

Abbreviations: N/A, not applicable; US, United States; UK, United Kingdom; BMI, body mass index; PwO, people with obesity; NHS: National Health System; WHO, World Health Organization; CDC, Centres of Disease Control and Prevention; EASO, European Association for Study of Obesity.
